# Environmental variability shapes the representational format of cultural learning

**DOI:** 10.1073/pnas.2505283122

**Published:** 2025-07-07

**Authors:** Xavier Roberts-Gaal, Marija Bolic, Fiery A. Cushman

**Affiliations:** ^a^Department of Psychology, Harvard University, Cambridge, MA 02138; ^b^Department of Psychology, University of Toronto, Toronto, ON M5S 3G3, Canada

**Keywords:** cultural evolution, social learning, model-free, model-based, environmental variability

## Abstract

Cumulative culture requires learning mechanisms that are both efficient and flexible in the face of environmental change. We examine models of this learning mechanism that emphasize teaching what to do (causally opaque procedures) and those that foreground what to aim for and why (goals and causal reasoning). Learning procedures is cheap but inflexible; learning goals is more flexible to changing circumstance, but requires expensive individual learning about how to achieve them. In an iterated learning experiment, we demonstrate that cultural learning adapts in precisely this way: Microcultures more often instruct future generations to follow procedures when the world is stable, but they tend to share information about valuable outcomes and causal relations when the world is variable.

Cumulative culture—improvement in artifacts, concepts, and practices over many generations—empowers people to acquire adaptive behaviors that no individual could invent from scratch (1–3). Currently, two models describe how this works. First, culture may encode causally opaque procedures, teaching us what to do, but not why it should be done (4, 5). For instance, some indigenous American cultures encode the processing of corn into masa as a traditional practice, but do not explain why this preparation is valuable (i.e., its nutritional benefits, 1). Second, culture may be intimately connected with causal reasoning. Culture may encode causal knowledge directly, such as scientific facts and principles, to help people reason how to accomplish their goals (1, 6). Culture may also rely on causal reasoning by encoding goals and values, and leaving it to individuals to figure out how they can best be realized (7).

Both models enjoy ample support. Culture sometimes tells us what to do, and other times what to aim for and why—a distinction embodied in the contrasting social learning strategies of imitation and emulation (2, 8). This motivates our question: What governs the form of cultural encoding that arises in a given context?

Our key insight involves a tradeoff between the cost of causal learning and its flexibility. Consider a world in which environments are stable: Across generations, the same actions consistently produce the best outcomes. In this case, encoding causally opaque procedures should be favored for its efficiency—it demands neither learning nor reasoning, but merely high-fidelity imitation. Now consider a world in which environments are variable: Here, the best actions change from generation to generation. In this case, encoding goals to emulate is necessary because individuals must discover for themselves the specific actions required to accomplish those goals.

As an illustrative example, suppose a fisherman can choose to use one of two baits (his actions), each of which yields some probability of catching one of two fish (his outcomes), each of which in turn yields some nutritional value (i.e., fitness). If the baits differ in their propensities to catch each type of fish, and if the fish differ in their fitnesses, then cultures should evolve to transmit adaptive information. Specifically, if both the causal relationship between bait and fish and the value of each fish are constant over time, encoding a causally opaque procedure that employs the optimal bait is most efficient. On the other hand, if the value of each fish remains constant, but the causal relationship between bait and fish varies across generations, encoding information about the valuable fish but requiring individuals to discover for themselves the bait that catches it would be favored for its flexibility.

Culturally learned procedures vs. goals resemble the well-studied distinction between model-free and model-based methods of reinforcement learning, respectively (9, 10). Model-free methods (which do not require causal learning) are computationally inexpensive but relatively inflexible in the face of environmental change. Model-based methods (which require causal learning) are more computationally demanding but can adapt more flexibly to changing environments (11). We propose that social learning, like individual learning, involves a tradeoff between flexibility and efficiency. This analogy guides our experiment.

## An Iterated Learning Experiment

**Fig. 1.**
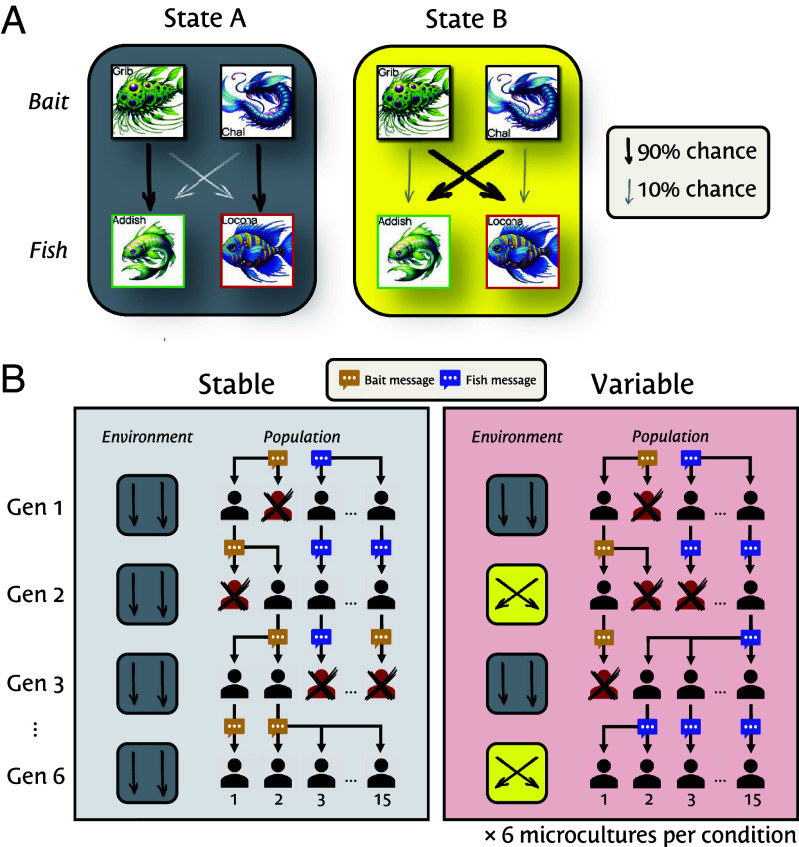
Experimental design and predictions. (*A*) Task schematic. On each of 12 trials, participants choose which bait to fish with (two of four bait shown). Each bait catches one fish with 90% probability and the other with 10%. In the stable condition, these mappings are constant across all generations within a microculture; in the variable condition, they reverse between every generation. (*B*) Iterated learning design. Participants (*n*= 1,093) are assigned to a condition (variable vs. stable), microculture within that condition (1 to 6, one shown), and generation within that microculture (1 to 6, n≈15 per generation). Each generation receives a message giving advice. Then, participants complete the task. Finally, participants can edit their message before transmitting it to the next generation. The probability a message is passed on is proportional to the fitness of the individual who left it, relative to their contemporaries in their microculture. Our predictions are illustrated.

We tested the predicted relationship between environmental variability and social learning in a large, online iterated learning experiment (12). Six generations of participants grouped in 12 “microcultures” (*n*= 1,093; ≈15 per microculture–generation) performed a fishing task like the one described above, and then left written advice for subsequent participants (a new “generation”; see Fig. 1). In the first generation, participants were seeded randomly with one of two messages: one recommending the optimal bait (“bait message”) and the other recommending the optimal fish (“fish message”). From the second generation onward, participants received advice from an ancestor in the prior generation of their microculture, sampled proportional to fitness. There were two conditions. In the stable environment, each bait tended to catch the same fish across generations. In the variable environment, each bait caught different fish in successive generations. The fitness of each fish was held constant. We predicted that in the stable condition, messages instructing which bait to use (procedures) will predominate, but in the variable condition, messages will more often instruct which fish to aim for (goal), leaving individuals to discover the appropriate bait for themselves. We coded messages for mentions of bait or fish names.

To isolate the influence of cultural evolution on the form of social learning, precluding results due to intelligent human design, we took three steps. First, we did not provide participants with any reliable information about the fitness of their behaviors (e.g., the quality of the fish they caught). Second, we did not inform them that the messages they received from prior generations were selected proportional to fitness. Third, we did not inform them about the possibility of environmental variability between generations. Thus, if the content of messages is affected by our manipulation of environmental variability, this should reflect cultural selection rather than intelligent design.

**Fig. 2.**
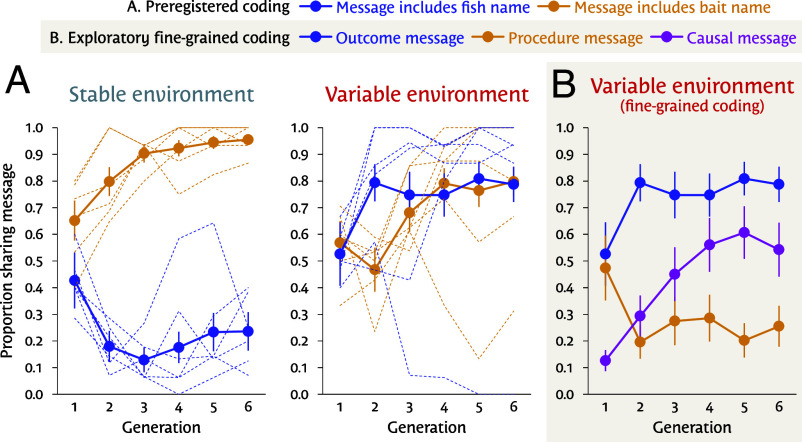
Key results. (*A*) Preregistered coding. Proportion of messages left by participants that included information about bait (orange) and about fish (blue) in the fishing task, by generation and by condition. Individual microcultures are plotted as faint lines. (*B*) Exploratory fine-grained coding. We examined how participants used bait terms in the variable condition. Outcome messages [blue, same as panel (*A*)] said which fish were valuable. Procedure messages (orange) recommended which bait to use, but not why. Causal messages (purple) used bait information to convey causal relationships. Note: Error bars indicate SEs clustered at the microculture level. Messages can include multiple types of information.

As Fig. 2*A* shows, microcultures in the stable condition tended to preserve messages about bait: 95.5% of messages in the final generation included bait terms, and only 23.6% included fish terms. In contrast, microcultures in the variable condition showed a greater tendency to include fish terms (78.7% in the final generation). We estimated a participant-level logistic mixed-effects model regressing the probability of transmitting information about bait on condition, with generation- and culture-level random intercepts. As predicted, participants’ transmitted messages in the variable condition were less likely to contain information about bait compared to those in the stable condition (b=−1.197, P=0.003, *OR*: 0.302, 95% CI: [0.127, 0.703]). An exploratory model showed messages in the variable condition were more likely to contain fish information than messages in the stable condition (b=2.471, $P=1.12\times 10^{-5}$, *OR*: 11.835, 95% CI: [3.601, 39.610]).

Contrary to our predictions, most messages in the variable condition also contained bait information (79.8% in the final generation). A finer-grained exploratory coding scheme revealed that most of these messages (68.0%) used bait information to convey a causal model of how to catch the valuable fish, not to recommend a bait (Fig. 2*B*). Indeed, this scheme showed that messages in the variable condition were more likely to report causal relationships than those in the stable condition (b=1.345, P=0.007, *OR:* 3.836, 95% CI: [1.323, 11.201]. But, due to environmental variability, causal messages were often out of date. Perhaps for this reason, while microcultures in the stable condition tended to show increasing fitness over generations in a logistic mixed-effects model of bait choice (b=0.081, P=0.03, *OR*: 1.085, 95% CI: [1.007, 1.170]), those in the variable condition did not (b=0.03, P=0.341, *OR*: 1.033, 95% CI: [0.966, 1.104]).

## Discussion

In an iterated learning task, microcultures of human participants converged on distinctive patterns of cultural transmission governed by environmental variability of which they were unaware. Specifically, in the variable condition, cultures adapted to pass messages about fish (what to aim for), enabling future generations to generalize effectively despite changes in the causal structure of the environment. In the stable condition, cultures predominantly passed messages simply about bait (what to do), bypassing unnecessary causal learning. Thus, we find that cultural evolution shapes the form of culturally transmitted information.

Unexpectedly, participants often shared bait information even in the variable condition. This occurred despite selective pressure, since outdated information about which bait to use likely led participants astray. (Perhaps for this reason, although we predicted that all cultures would improve in fitness over six generations, only cultures in the stable condition did so.) Inspecting these messages more closely, most of them employed bait information not to bypass causal representation, but to facilitate it; for example, one participant noted “Zigu [bait] seems to consistently catch Addish fish in my experience.” Even in the variable condition, any individual participant experienced stability within their own lifetime, and thus may have augmented a fish-only message with bait information in an attempt (unwittingly misguided) to facilitate causal reasoning in the subsequent generation. This apparent interplay between evolutionary pressures and psychological predispositions or constraints (e.g., learnability, 13, 14; convergence to a cultural attractor, 15) stands out as an important area for further research. Cultural selection and individual cognition can work at cross purposes, or in concert, to drive cultural evolution.

Unlike many prior studies of iterated learning (see ref. 12), we prevented participants from observing the fitness of their actions while using fitness ourselves to bias the transmission of messages. These design choices enabled us to experimentally isolate the effect of cultural evolution on the content of participants’ messages. In natural contexts, however, people often have direct knowledge of the fitness of their actions. Future work should explore how people use this knowledge to purposefully shape the information encoded by culture.

Some current theories of cumulative culture highlight high-fidelity imitation of causally opaque practices (4), while others emphasize on-the-fly causal reasoning (6). The present work provides an angler’s guide for understanding the trade-offs between these mechanisms. We observe that environmental variability can induce iterated learning chains to adapt the format of cultural transmission, as it establishes the optimal balance between flexibility and efficiency in social learning. This may be an important determinant of the differences between human cultures in their tendency to transmit causally opaque practices vs. values and causal knowledge.

## Materials and Methods

Materials, data, analysis scripts, and preregistration documents can be found on OSF (16). Detailed design information is available in *SI Appendix*. This study was approved by the Harvard University-Area Institutional Review Board (protocol #IRB14-2016). All participants provided informed consent.

1,110 participants were recruited via Amazon Mechanical Turk using CloudResearch. 17 (1.53%) failed an attention check, yielding a final sample of 1,093 (59.2% women, 39.8% men, 1.0% other; age M = 43.3, sd = 13.1). Participants were randomly assigned to a microculture in one of two conditions (stable vs. variable environment) and were sequentially assigned to one generation within that microculture. We established six microcultures per condition of six generations each, comprising approximately 15 participants per generation (12 to 18 due to randomization).

After instructions describing the fishing task, participants were presented with a message from a participant in the prior generation of the same microculture (or, in the first generation, a seeded message as described in the main text). They then completed 12 trials. On a trial, participants chose a bait to use, observed the fish they caught, and observed the price that the fish obtained in the marketplace (drawn from a noisy truncated normal distribution). We included market prices because we found in pilot testing that they increased participant engagement; however, they carried no useful information about the fitness of bait choices. Finally, participants were shown the message they saw previously and offered the opportunity to edit it for future participants. We transmitted participants’ messages to the next generation with probability proportional to their performance at picking the right bait (the bait that tend to catch the highest fitness fish in that generation), compared to those in the same generation and culture.

Half of the microcultures continued fishing and dispensing advice in a stable environment where the same bait almost always caught the same fish, while the remainder faced a highly variable environment, where each bait tended to catch different fish in successive generations.

## Supplementary Material

Appendix 01 (PDF)

Dataset S01 (CSV)

Dataset S02 (CSV)

## Data Availability

Anonymized participant data (.csv) materials, and software have been deposited in OSF (https://osf.io/p8abj/) (16). All data are also included in the manuscript and/or supporting information.
