# Supplementary material for: Environmental variability shapes the representational format of cultural learning
Source: Proc Natl Acad Sci U S A. 2025 Jul 7;122(28):e2505283122. doi: 10.1073/pnas.2505283122 (PMC12280920; doi:10.1073/pnas.2505283122)
Supplement: Supplementary file 1 — Appendix 01 (PDF) [file pnas.2505283122.sapp.pdf]

Supporting Information for

Environmental variability shapes the representational format of cultural learning

Xavier Roberts-Gaal, Marija Bolic, and Fiery Cushman.

Xavier Roberts-Gaal
xavierrobertsgaal@g.harvard.edu

This PDF file includes:

Supporting text
Legends for Dataset S1 to S2
SI References

Other supporting materials for this manuscript include the following:

Datasets S1 to S2

Supporting Information Text

Extended methods

Overall design. Participants received a message instructing them how to successfully play a social learning game, described in detail below. Then, participants completed 12 trials of the game and passed on a message of their own before completing demographic items and questionnaires. We assigned participants sequentially to each of six generations. Within each generation, we randomly assigned participants to a 2 (condition: stable vs. variable environment) $\times$ 6 (microculture) design, between-subjects. Owing to randomization, cell sizes by generation, condition, and microculture varied slightly: the smallest cell size was 12 and the mean cell size was 15.2. All six generations had ≥ 179 participants.

Task structure. Participants were instructed that they have recently moved to Misthaven, a fishing community abutting a fog-shrouded lake. They received advice from an expert fisher, who handed them a free-text message in the form of a scroll containing fishing wisdom.

Participants then played 12 trials of a modified two-step task (1): they picked one of four kinds of insect (named with pseudowords: Grib, Zigu, Chal, Vlid) to use as fishing bait. Two fish types were available (Addish and Locona). Each insect always caught exactly one fish, but the identity of the fish depended on the insect and the hidden world state (A or B). The transition probabilities for the fishing task were as follows:

- In world state A, Grib and Zigu bait caught Addish with probability 0.9 and Locona with probability 0.1
- In world state A, Chal and Vlid bait caught Addish with probability 0.1 and Locona with probability 0.9
- In world state B, Grib and Zigu bait caught Addish with probability 0.1 and Locona with probability 0.9
- In world state B, Chal and Vlid bait caught Addish with probability 0.9 and Locona with probability 0.1

Participants learned that each bait cost 25 in-game coins. Fish could be sold for coins. The price of a fish (i.e., reward) was sampled from a truncated normal distribution with limits $[0, 60]$, sd 20, and mean of either 24 (Locona) or 26 (Addish). These distributions were carefully chosen to be nearly impossible to accurately distinguish after only 12 trials. Each day, participants saw that day's fish sale and a running coin count.

Critical manipulation: In the stable condition, the world always remained in state A. In the variable condition, the world switched state before each generation (including generation 1).

After playing 12 trials, participants were asked to pass on the advice scroll to a new arrival in town. They had the opportunity to edit the scroll's message (total message length up to 500 characters) or pass it down unchanged. Between generations, we screened out any sentences containing profanity or vulgarity, consistent with our preregistered procedure. As indicated in our data dictionary, data file, and code, only one sentence from one message in generation four was screened out for vulgarity.

Cultural selection and messages. In the first generation, participants received one of two seed messages with equal probability. One seed message was designed to support learning the correct action ("A successful fisherman absolutely must pick the right bait to use. Grib and Zigu bait are the best"), while the other was designed to support learning the most valuable fish ("A successful fisherman absolutely must catch the fish that fetches the highest price. Addish fish will earn you the most. Everyone needs to figure out for themselves which bait is most successful at catching Addish").

In subsequent generations, participants received a message from a player in the previous generation of their microculture. Transmitted messages were passed along with probability determined by the softmax of the transmitter's fitness, relative to the fitness of each participant belonging to the same microculture and generation. A participant's fitness was calculated as the *number of trials* on which they chose correctly—that is, picked one of the bait types that were most likely to yield Addish fish. For example, in state A, a player picking a Grib or a Zigu would have received +1 fitness, while a player choosing a Chal or a Vlid would have received +0. We chose this selection mechanism for three reasons. First, it reduces noise in evolutionary dynamics over selection based on the number of Addish fish caught, since our environment included stochastic transitions. Second, it better models fitness-conferring activities with opaque or noisy hedonic values, such as eating optimally healthy vs. slightly vitamin-deficient food. Third, it decorrelates a fish's observed reward (its sale price at market) from its unobserved fitness benefits.

Screenshots of experimental materials are available on OSF.

Analysis strategy. To analyze participants' messages, we applied a transparent, preregistered coding scheme to generate two binary-valued scores per message. We coded a message as a "bait message" if it contained the name of one of the bait types (from which participants could learn a procedure). We coded a message as a "fish message" if it mentioned a fish type by name (straightforwardly supporting goal learning). As preregistered, we report results only from codings using exact matches for fish and bait names. However, our results are also robust to fuzzy matches (Levenshtein distance = 1).

After conducting our preregistered analysis, we wanted to better understand how messages employed bait information—for example, in the variable condition, we wanted to disambiguate whether "bait messages" were using bait terms to recommend a causally opaque procedure, or instead to convey causal relationships. Therefore, we re-analyzed our data with an exploratory finer-grained coding scheme. Using this scheme, we coded messages blind to condition (variability, microculture, and generation).

We coded a message as an "outcome message" if it mentioned a fish type by name to support goal learning. We coded a message as a "causal message" if it mentioned a bait type and a fish type in order to convey causal relationships between bait and fish. Example causal messages in the variable condition included:

- "A successful fisherman absolutely must catch the fish that fetches the highest price. Addish fish will earn you the most. Everyone needs to figure out for themselves which bait is most successful at catching Addish, but Zigu [bait] seems to consistently catch Addish fish in my experience."
- "A successful fisherman absolutely must catch the fish that fetches the highest price. Addish fish will earn you the most. Everyone needs to figure out for themselves which bait is most successful at catching Addish, but Zigu [bait] seems to consistently catch Addish fish in my experience."
- "A successful fisherman absolutely must catch the fish that fetches the highest price. Addish fish will earn you the most. Everyone needs to figure out for themselves which bait is most successful at catching Addish. Vlid have been known to be bait that can attract Addish. Chal also have been known to be bait that attract Addish."
- "A successful fisherman absolutely must catch the fish that fetches the highest price. Addish fish will earn you the most. Everyone needs to figure out for themselves which bait is most successful at catching Addish. For me, Zigu is the best bait to attract Addish. There are other baits that work to catch Addish fish. Figure out for yourself which one works best for you."
- "A successful fisherman absolutely must catch the fish that fetches the highest price. Addish fish will earn you the most. Everyone needs to figure out for themselves which bait is most successful at catching Addish. Grib [bait] has been known to catch Addish."

We coded a message as a "procedure message" if it mentioned a bait type by name, recommending using it without drawing a causal connection to the fish it caught. The exploratory coding scheme was also robust to whether we corrected for spelling errors (i.e., exact matches vs. Levenshtein distance = 1).

Messages in both the preregistered and the exploratory coding schemes could contain multiple types of information. For example, this message from the variable condition was coded as an outcome message and a procedure message (but not a causal message) using our exploratory fine-grained coding scheme: "A successful fisherman absolutely must pick the right bait to use. Grib and Zigu bait are the best. Best fish to get is Addish."

Statistical methods. To analyze participant behavior, we fit several preregistered trial-level and participant-level logistic mixed-effects models using the `lme4` package (2). Motivated by the dependence of participants' behavior on their microculture (learning chain) and the previous generation, we included appropriate random intercepts (microculture-, generation-, and participant-level where possible). To interpret the resulting predictions as increasing the odds of behavior and allow for learning effects, we did not fit random slopes; however, results from models with maximal random effects converged qualitatively. We describe models in the order in which they appear in the main text.

First, we fit a logistic model at the participant level testing whether a participant is more likely to pass along a bait message in the no-shocks condition than in the shocks condition, with generation- and microculture-level random intercepts. This model was specified as:

$$\text{Bait Message} \sim \text{Condition} + (1|\text{Generation}) + (1|\text{Culture})$$

We also fit an exploratory fish message model, replacing the bait message indicator variable with a fish message indicator. And, we fit an exploratory model using our fine-grained coding scheme (i.e., with a causal message indicator).

Second, we fit logistic models at the trial level separately to data from each condition, predicting whether a participant would take a fitness-enhancing action (selecting one of the two correct bait types, given the world state) by their generation, with participant- and microculture-level random intercepts. The `glmer`-style model specification is:

$$\text{Correct Bait} \sim \text{Generation} + (1|\text{Culture}) + (1|\text{Participant})$$

All analysis code is available on OSF.

Variables collected for exploratory purposes. In addition to the main variables used in preregistered analyses, and standard demographics (age, gender, income, education), we also collected several exploratory measures:

- **Explicit causal understanding:** Number of questions correctly answered about which fish each bait tends to catch (0-4). Correct answers were set by the generative causal model of the experiment.
- **Response time:** Time taken to select a bait in milliseconds (per trial and cumulative).
- **Self-report ancestry:** Participants answered one question: "We are interested in understanding ancestry and cultural origin. Five hundred years ago, in which countries did your ancestors live?" Participants were instructed to select at least one geographic region corresponding to a present-day country/territory, and could select multiple.
- **Urbanicity:** Self-report item assessing the degree to which a participant's childhood was in a rural/urban area.

- **Traditionalism:** One item (V79) from the World Values Survey Wave 6 (3). Text: "How similar is this person to you? Tradition is important to this person. They think it's important to follow the customs handed down by one's religion or family."

- **Relational mobility:** Five items from the relational mobility scale (4), with wording changed to refer to one's own community ("How well do the following statements describe your community?"), namely: "You are able to choose, according to your own preferences, the people you interact with in your community", "If you did not like your current group(s), you could leave for better ones", "It is easy for you to meet new people", "You are able to choose the groups and organizations you belong to", and "There are few opportunities for you to form new friendships" (final item is reverse-scored).

- **Moral beliefs:** Oxford Utilitarianism Scale (5).

- **Attitudes toward obedience:** One item querying the extent to which a participant believes children should be obedient vs. independent.

We do not report analyses of these exploratory variables here, but raw data are available on OSF.

SI Dataset S1 (environmental_variability_study.csv)

Data analyzed for the study. This is also available on OSF.

SI Dataset S2 (messages_shuffled_CODED.csv)

Participant messages, coded using an exploratory coding scheme as described above. This is also available on OSF.

References

1. ND Daw, SJ Gershman, B Seymour, P Dayan, RJ Dolan, Model-Based Influences on Humans' Choices and Striatal Prediction Errors. *Neuron* **69**, 1204–1215 (2011).
2. D Bates, M Mächler, B Bolker, S Walker, Fitting Linear Mixed-Effects Models Using lme4. *J. Stat. Softw.* **67**, 1–48 (2015).
3. R Inglehart, et al., eds., *World values survey: Round six - country-pooled datafile version.* (JD Systems Institute, Madrid), (2014).
4. R Thomson, et al., Relational mobility predicts social behaviors in 39 countries and is tied to historical farming and threat. *Proc. Natl. Acad. Sci. United States Am.* **115**, 7521–7526 (2018).
5. G Kahane, et al., Oxford Utilitarianism Scale (2018) Institution: American Psychological Association.
